# The fabrication of an ICA-SF/PLCL nanofibrous membrane by coaxial electrospinning and its effect on bone regeneration *in vitro* and *in vivo*

**DOI:** 10.1038/s41598-017-07759-8

**Published:** 2017-08-17

**Authors:** Lihua Yin, Kaijuan Wang, Xiaoqin Lv, Rui Sun, Shaohua Yang, Yujie Yang, Yanyun Liu, Jiatao Liu, Jing Zhou, Zhanhai Yu

**Affiliations:** 1 Department of Oral Implantology, School/Hospital of Stomatology, Lanzhou University, Lanzhou, China 730000; 2 School/Hospital of Stomatology, Lanzhou University, Lanzhou, 730000 China

## Abstract

GBR is currently accepted as one of the most effective approaches for bone defect regeneration relating to dental implant. Icariin is the main active ingredient in the extraction of total flavonoids from the Chinese traditional herb *Epimediumbrevicornum* Maxim. In this study, ICA was successfully incorporated into the nanofibers barrier membrane (ICA-SF/PLCL) as osteoinduction factor by coaxial electrospinning and was released in a sustained and controlled manner. The entire release period included two stages: an initial burst stage (47.54 ± 0.06% on 5 d) and a decreasing and constant stage (82.09 ± 1.86% on 30 d). The membrane has good biocompatibility with BMMSCs anchored and significantly promoted its osteogenic activity. Moreover, *in vivo* experiment, bone defect covered by ICA-SF/PLCL membrane in rat cranium were statistically repaired compare to other groups. 12 weeks after implantation, in the test group, the new bone formation spread to cover most of the defect region with volume and density of approximately 15.95 ± 3.58 mm^3^ and 14.02 ± 0.93%. These results demonstrated that ICA-SF/PLCL nanofibrous membrane could be a promising barrier applicated for GBR.

## Introduction

Dental implants have become increasingly accepted as the preferred method for the treatment of missing teeth^1^; however, bone deficiency caused by inflammation, as can occur with granulation tissue, can threaten implant success rates^2^. A growing number of studies have shown that guided bone regeneration (GBR) technology can effectively restore the height and fullness of the alveolar bone, which can have a barrier function by preventing fibroblast cells and epithelial cells ingrowth into the bone defect site, and increase bone regeneration by increasing osteoblast attachment and proliferation^3–5^.

A variety of biological materials currently on the market are applied in GBR, including non-biodegradable polytetrafluoroethylene (expanded polytetrafluoroethylene), which requires a second surgery to remove, so that infection and membrane exposure are risks^5, 6^. Many researchers have thus focused on biodegradable membranes. Studies have shown that Bio-Gide® is a conventional biodegradable membrane with good biocompatibility, low antigenicity, sufficient mechanical properties, and a proper degradation profile^7^. But, all barrier membranes commercially available lack of osteoinduction which will greatly promote new bone regeneration and bone remodeling. Now, a composite functional GBR membrane is desired. The point is to combine the osteoinductive factors with the biodegradable materials in a biocompatible way and keep the inducer released sustained and controlled.

Icariin (ICA; C_33_H_40_O_15_; molecular weight: 676.67) is the main active ingredient in the extraction of total flavonoids from the Chinese herb *Epimediumbrevicornum* Maxim^8^. ICA has many important physiological activities, such as promoting the proliferation and differentiation of osteoblasts to protect against metabolic bone disease, along with immune regulation and anti-tumor activity^9^. Studies have found that ICA can treat bone loss in postmenopausal women and restore bone strength associated with bone marrow stromal cell differentiation into osteoblasts via enhanced expression of osteoprotegerin and bone morphogenetic protein^10^. Ultimately, ICA significantly inhibits osteoclast formation^11^; therefore, it is considered an osteogenic inducer for bone tissue engineering. Our previous studies have found that ICA can significantly promote the proliferation and osteogenic differentiation abilities of human periodontal ligament stem cells both *in vitro* and *in vivo* ^12^.

Coaxial electrospinning is a simple and effective method for preparing scaffolds with nanometer or submicron fibers, which has gained broad application in tissue engineering and drug delivery systems^13^. The coaxial electrospinning scaffolds with a high surface-to-volume ratio can promote cell attachment, achieve drug loading, and demonstrate sustained and controlled local drug delivery^14^. Previous studies have shown that some biomolecules can be successfully encapsulated in the nanofibers while the biomolecule bioactivity is retained and that the diameter of the fibers can be controlled by tailoring parameters in coaxial ectrospinning technology. Thus, in this study, we fabricated the ICA-SF/PLCL nanofibrous membrane by coaxial electrospinning.

Silk fibroin (SF) is a natural structural protein with good biological characteristics, such as good cell adhesion, controllable degradation, mechanical strength, permeability, and resistance to enzymatic degradation^15^. It has been extensively researched for tissue engineering and controlled drug delivery systems^16, 17^. Thus, the ICA-SF/PLCL coaxial electrospinning nanofibrous membrane was used for the GBR membrane in this study. Although various studies of GBR membrane promotion of bone regeneration have been conducted^18–21^, the combination of GBR technology, coaxial electrospinning technology, and growth factors to promote bone regeneration has rarely been reported. Research have found that rhBMP-2 as a growth factor could be successfully contained in a coaxial electrospinning nanofibrous membrane, which realized sustained drug-release and preserved biological activity. The membrane served as not only a GBR membrane *in vivo* but also a biodegradable scaffold for tissue engineering^22^.

The objective of this study was to develop an osteoinductive membrane used in GBR to promote bone regeneration with sustained release of ICA. The parameters during the coaxial electrospinning process were confirmed by our previous work^23–26^, and the physical and mechanical properties, degradation, drug release, and effect on bone regeneration *in vitro* and *in vivo* were evaluated.

## Materials and Methods

### Preparation of coaxial electrospinning nanofibrous membrane

*Bombyx mori* cocoons (Jiaxing Silk Co., Ltd., Jiaxing, China) were degummed in 0.5% (w/v) Na_2_CO_3_ solution at 100 °C twice for 1 h, washed with distilled water three times to remove sericin, and then dried at 50 °C for 12 h. After being dissolved completely in a ternary solvent system of CaCl_2_/CH_3_CH_2_OH/H_2_O (mole ratio 1/2/8) at 80 °C, the SF solution was dialyzed in ultrapure water for 3–5 d at room temperature and filtered and freeze-dried for 24 h to obtain regenerated SF sponges. After that, the SF/PLCL (weight ratios 30:70; PLCL, M_W_ = 1,000,000; provided by Nara Medical University, Japan) was dissolved in HFIP (Daikin Industries, Ltd., Japan) at room temperature at a concentration of 8 w/v% under magnetic stirring for 6 h. The obtained solution was used as the shell layer solution, and 10^−5^ mol/L ICA (Bio-function Co., Ltd., Beijing, China, according to our previous study^23–25^ was used as the core layer solution. The two solutions were placed in two 10 mL syringes with a high-voltage power supply (15 kV; DW-P503-1ACDF; Beijing, China). For the advancing velocities of the shell and core, 1.0 mL/h and 0.1 mL/h were chosen, respectively. Aluminum foil was used as a collector that was 15 cm away from the needle. All electrospinning processes were carried out at room temperature with a relative humidity of 50 ± 6%. The nanofiber membrane lacking ICA (SF/PLCL membrane) was similarly fabricated as a control. The membranes were then crosslinked by glutaraldehyde (GTA; Sinopharm Chemical Reagent Co., Ltd., China) solution (25%) for 48 h and vacuum dried.

### Characterization of the ICA-SF/PLCL nanofibrous membrane

In this section, we used 7 methods to evaluate the physical, chemical and mechanical properties^26, 27^.

#### Scanning electron microscopy (SEM)

The samples (3 × 3 mm) of ICA- SF/PLCL and SF/PLCL for SEM were vacuum dried and coated with gold for 60 s before observation (JSM-4800; JEOL, Tokyo, Japan) at a voltage of 10 kV. The fiber diameters were measured at 100 random locations (Image J software, National Institutes of Health, MD, USA).

#### Transmission electron microscopy (TEM)

The core–shell structure of the nanofibers was verified at 100 kV (TEM: FEI Tecnai F3, USA). The samples of SF/PLCL and ICA- SF/PLCL were prepared by depositing the fabricating fibers onto copper grids followed by vacuum drying for 7 d prior to TEM imaging.

#### X-ray diffraction (XRD) pattern

XRD analysis was conducted to determine the crystal structure of matter. The curve was obtained on an X-ray diffractometer (D/Max-2400; Rigaku, Tokyo, Japan) with a scanning speed of 6°/min at 40 kV/150 mA.

#### Contact angle measurement

The water contact angle measurement was used to test the hydrophilicity of the membrane. A contact angle metering system (developed by the College of Chemistry and Chemical Engineering, Lanzhou University, China) was used to evaluate the wettability of the ICA-SF/PLCL nanofibrous membrane. After 0.03 mL of distilled water was dropped carefully onto the membrane (20 × 30 mm) surface, the angles between the water droplet and the membrane surface were measured after 5 s. Five sites were tested for each sample, and the average value with standard deviation is reported.

#### Tensile strength tests

The mechanical properties of the ICA-SF/PLCL nanofibrous membrane were tested using a universal material tester (ANS CMT8102, Hounsfield, UK). Five samples (10 × 30 mm) fixed with two clamps were tested, and the stress–strain curve was developed under application of a 10 N tensile load and tensile speed of 1 mm/min. The mean values are presented.

#### Drug release profiles *in vitro*

All of the squared membranes were sterilized with ultraviolet light for 1 h on each side and immersed in 30 mL phosphate-buffered saline (PBS; 0.05 M, pH 7.4). The test was performed on a shaking incubator at 37 °C at 100 rpm^23^. At time points of 1, 2, 3, 4, 5, 10, 15, 20, 25, and 30 d, 5 mL of medium was taken out to determine ICA concentrations (ë_max_ = 270 nm) using a UV–vis spectrophotometer (UV 2600, Shimadzu, Japan), and was refilled with another 5 mL of fresh PBS. The amount of ICA released from the ICA-SF/PLCL nanofibrous membrane was calculated and the cumulative release curve was generated.

#### Biodegradation of the nanofibrous membrane *in vitro*

The samples (10 × 10 mm) were soaked in 20 mL PBS (pH 7.4) on a shaking incubator at 37 °C at 100 rpm. The morphology and tensile strength of the samples during degradation process were measured by SEM and tensile strength tests, respectively.

### ***In vitro*** experiments

#### Isolation, culture and id﻿﻿entification of BMMSCs

The bone marrow mesenchymal stem cells were isolated from Sprague–Dawley (SD) rats (female, age 3–4 weeks, 80–100 g, from the Experimental Animal Center of Lanzhou University). The primary BMMSCs were isolated and cultured according to a previously reported protocol with minor modifications^24^. Cells were incubated in low-glucose Dulbecco’s modified Eagle’s medium (L-DMEM) (HyClone, USA) containing 10% fetal bovine serum (FBS) and 100 U/mL penicillin/streptomycin (Gibco) at 37 °C under 5% CO_2_. Half of the liquid volume was renewed after 24 h and completely replaced after 48 h, followed by medium replacement every 48 h. The unattached cells were discarded by refreshing the medium, and cells of passage 3 were used for identification ﻿(Table S1, Fig. S1) and the subsequent experiments.

#### The activity of released ICA on osteogenic differentiation of BMMSCs

The grouping of this experiment is: i) Test group, BMMSCs cultured in ICA-SF/PLCL membrane–released medium group, ii) Control group, BMMSCs cultured in normal culture medium group, and iii) Blank control (no cells group). P3 BMMSCs were seeded onto plates at a density of 1.0 × 104 cells/mL and the culture medium containing released ICA from the membranes with concentration of 10^−6^ mol/L was added. The control group was cultured with normal medium. Then, the BMMSCs were cultured at 37 °C with 5% CO_2_ without passage and the medium was changed every 48 h. At 14 d after culturing, the P3 cells were fixed with 4% paraformaldehyde and stained with 2% Alizarin Red S (Genmed Scientifics Inc., USA) for 30 min at 37 °C for detection of calcium accumulation.

At time points of 3, 7, and 14 d, respectively, BMMSCs were lysed by 0.1% TritonX-100 (Ambion, by Life Technologies) and the intracellular ALP activity measured by p-nitrophenyl phosphate using an ALP assay kit (Nanjing, Jiancheng Bioengineering Institute, Nanjing, China) according to the provided instructions. The absorbance was determined at 520 nm using an enzyme-linked immunosorbent assay reader (Elx 800, Bio-Tek, Winooski, VT, USA), and data were normalized for total protein content using Bradford’s law.

#### Seeding and morphology of BMMSCs on the membranes

Before BMMSCs (passage 3, got from 2.3.1) were seeded onto the ICA-SF/PLCL nanofibrous membranes at a density of 1.0 × 10^4^ cells/well in 24-well plates under the pressure of a metal loop, the membranes (10 × 10 mm) were sterilized with ultraviolet light for 1 h on each side, washed with PBS three times, and then immersed in complete culture medium (L-DMEM containing 10% FBS and 100 U/mL penicillin/streptomycin) at 37 °C overnight. The cell–membrane constructs were incubated at 37 °C under 5% CO_2_ and the medium replaced every 48 h. After 5 d, the constructs were washed with PBS three times, fixed with 2.5% GTA for 1 h, dehydrated in a series of graded ethanol (30%, 50%, 70%, 90%, 95%, and 100%, 1 h each), and dried under vacuum. The dried samples were sputter-coated with gold for 60 s and observed by SEM (S-3400N; JEOL, Tokyo, Japan).

#### Cytotoxicity testing of the ICA-SF/PLCL nanofibrous membrane

The cytotoxicity of the membrane to BMMSCs was evaluated by the MTT assay (Sigma, USA)^23^. BMMSCs (1.0 × 10^4^ cells/well) were seeded onto 96-well plates and incubated overnight in L-DMEM containing 10% FBS, and the membranes (5 × 5 mm) were added to each well. At the time points of 1, 3, 5, and 7 d, 20 μL MTT solution (5 mg/mL in PBS, pH 7.4) was added instead of the medium, and the membranes were discarded. Then, 150 μL dimethyl sulfoxide (Sigma, USA) was added to each well and the assay processed after dissolution of crystals by oscillation for 30 min. Cells in complete culture medium with no membrane were used as control. Six parallel wells were used for each group. The results were detected by an enzyme-linked immunosorbent assay reader (Elx 800, Bio-Tek, Winooski, VT, USA) at 490 nm.

### In vivo implantation

#### Surgery procedure

**Figure 1:**
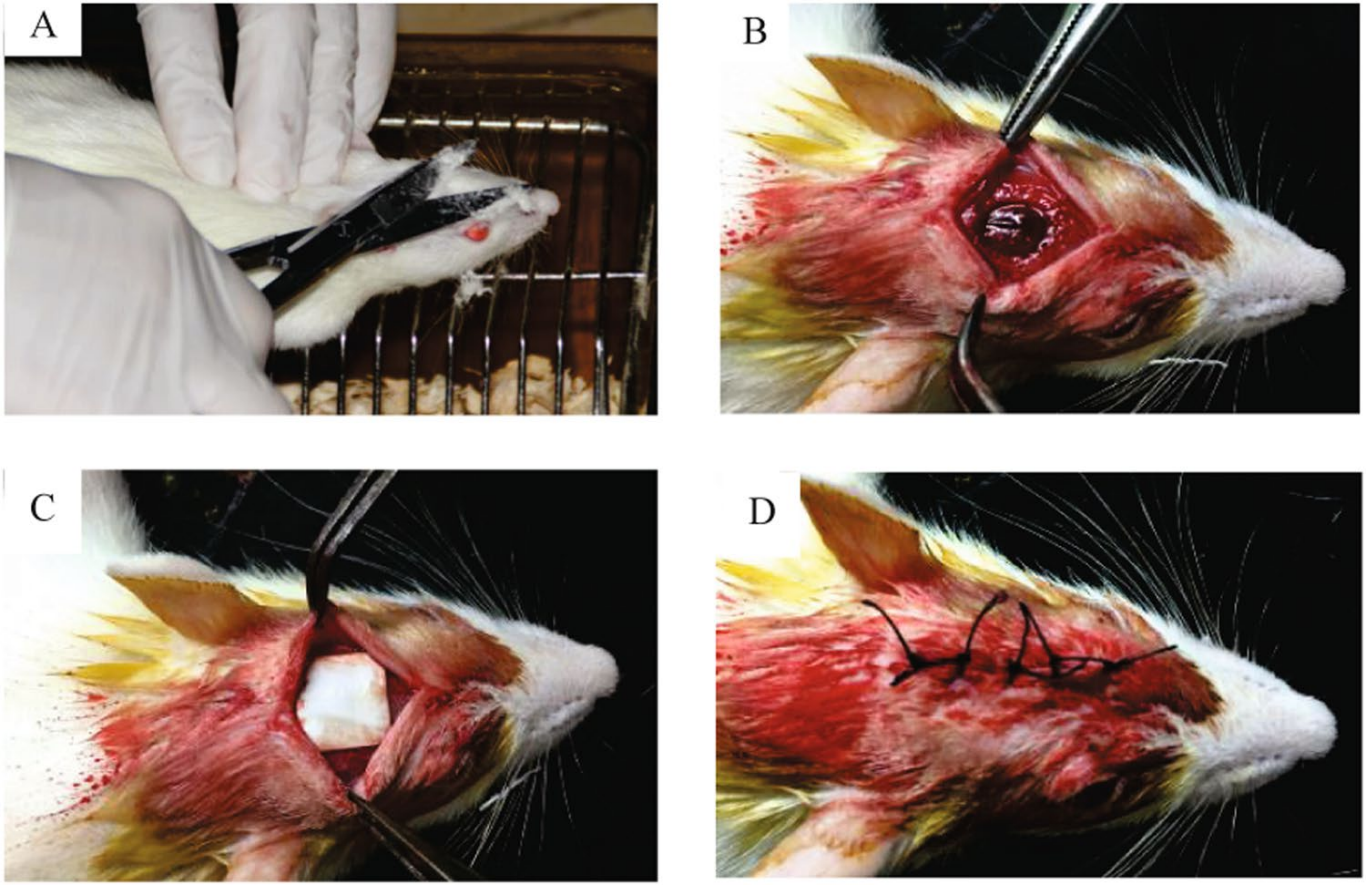
The animal experiment surgery procedure. (**A**) The preparation of SD rats for membrane implantation. (**B**) A complete defect was created on the cranium. (**C**) Implanting of the membrane and complete coverage of the defects. (**D**) Repositioning of the soft tissues and suturing layer by layer.

Twenty-seven healthy adult male SD rats were randomly divided into three groups, (i) test group (defect covered by ICA-SF/PLCL nanofibrous membrane, n = 3); (ii) negative control group (defect covered by SF/PLCL membrane, n = 3); (iii) and blank control group (defect covered with no membrane, n = 3). All surgeries were carried out aseptically, as follows: After the animal was placed under general anesthesia with 10% chloral hydrate (0.35 mL/100 g body weight), a mid-sagittal incision through the skin was created, the forehead was shaved and disinfected with iodine scrubs, and a full-thickness flap was elevated to expose the cranium completely. Then, by using a slow-speed dental drill (ROTEX782E, DENTAMRICA, USA) with abundant cool saline, a complete round defect 8 mm in diameter was created where the membranes (12 × 12 mm) were implanted and the defect was fully covered. The surgery region then was washed with normal saline and the soft tissue was repositioned before suturing layer by layer to achieve primary closure. After surgery, all animals were allowed to recover on a warm sheet and then transferred to the vivarium for postoperative care (Fig. 1).

#### Micro-computed tomography (μ-CT) evaluation

At the time points of 4, 8, and 12 weeks post-surgery, animals were euthanized and the calvarial tissues harvested and fixed in 4% paraformaldehyde. The samples then were evaluated using an animal μ-CT scanner (Quantum FX, PerkinElmer, Hopkinton, MA, USA) in high-resolution scanning mode at 90 kVp, 200 μA, field of view 72 mm, and 4.5 μm resolution. The images and data were analyzed using the μ-CT image analysis software (Skyscan, Kontich, Belgium), including measures of the mean volume of new bone and the bone mineral density.

#### Histological analysis

All fixed samples were decalcified, dehydrated, embedded in paraffin, and sliced at 4 μm thick parallel to the sectioned surface. Tissue sections were stained with hematoxylin and eosin (Nanjing Jiancheng Biotechnology Institute) for morphological evaluation and observed by optical microscopy (Olympus, IX 70, Japan). Areas of newly formed bone in each of the measured locations were quantified by using Image J software (National Institutes of Health, MD, USA), and the ratio of new bone area to the total defect area also was calculated.

### Statistical analysis

Statistical analysis was performed using SPSS ver. 17.0 software. All quantitative data are expressed as mean ± standard deviation. Statistical differences among the groups were assessed by one-way analysis of variance (ANOVA). A p value < 0.05 was considered statistically significant.

Institutional Animal Care Committee of School of Stomatology in Lanzhou University has approved all the methods above for the care, maintenance, and treatment of animals in the present study. Furthermore, all experiments were performed in accordance with relevant guidelines and regulations.

## Results

### Characterization of the ICA-SF/PLCL nanofibrous membrane

#### SEM

**Figure 2:**
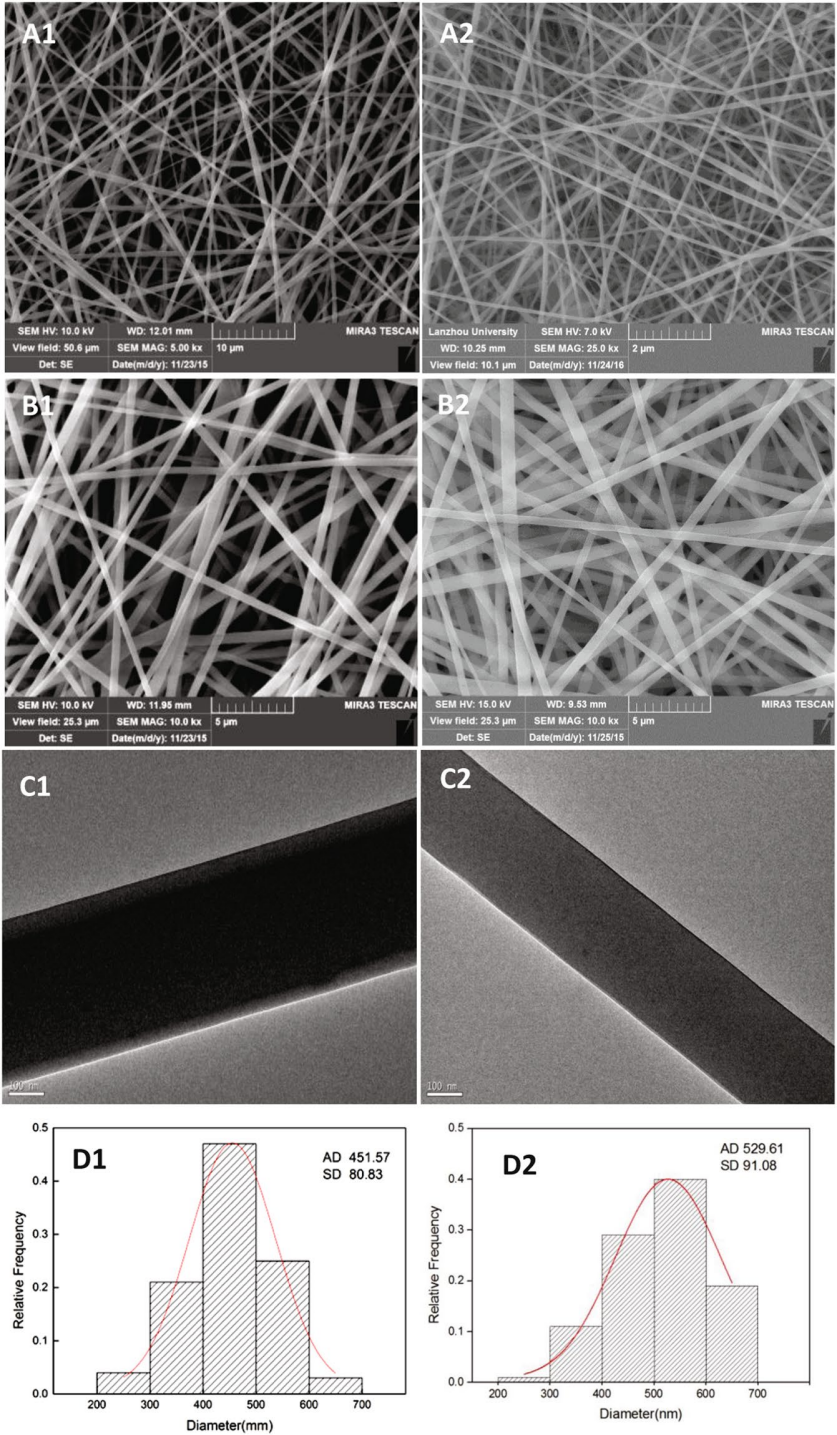
Representative SEM and TEM micrographs of the ICA-SF/PLCL and SF/PLCL nanofibrous membrane (scale bars: A1, 10 μm; A2, 2 μm; B1, B2, 5 μm; C1,C2, 100 nm) and (**D**) the diameter distribution histograms.

The SEM images of ICA-SF/PLCL and SF/PLCL nanofibrous membranes (Fig. 2A1,A2,B1,B2) showed uniformly distributed fibers with continuous, smooth, and homogeneous porous structures with no beads formed. As was shown in Fig. 2C1, the ICA-SF/PLCL fibers exhibited a typical core-shell structure while the fibers in SF/PLCL did not (Fig. 2C2). Furthermore, the mean diameter of the fibers in ICA-SF/PLCL group was 451.57 ± 80.33 nm, ranging from 200 to 700 nm (Fig. 2D1), and that in SF/PLCL group was 529.61 ± 91.08 nm, ranging from 200 to 700 nm (Fig. 2D2), respectively, which meant the morphology and the nano-scale structure were not changed by loading ICA.

#### TEM

The TEM images of ICA-SF/PLCL nanofibrous membranes (Fig. 2C) showed that a distinct boundary along with the fiber axis could be clearly observed, demonstrating the formation of a core–shell structure, indicating that ICA was successfully incorporated into the nanofiber by coaxial electrospinning.

#### XRD pattern

**Figure 3:**
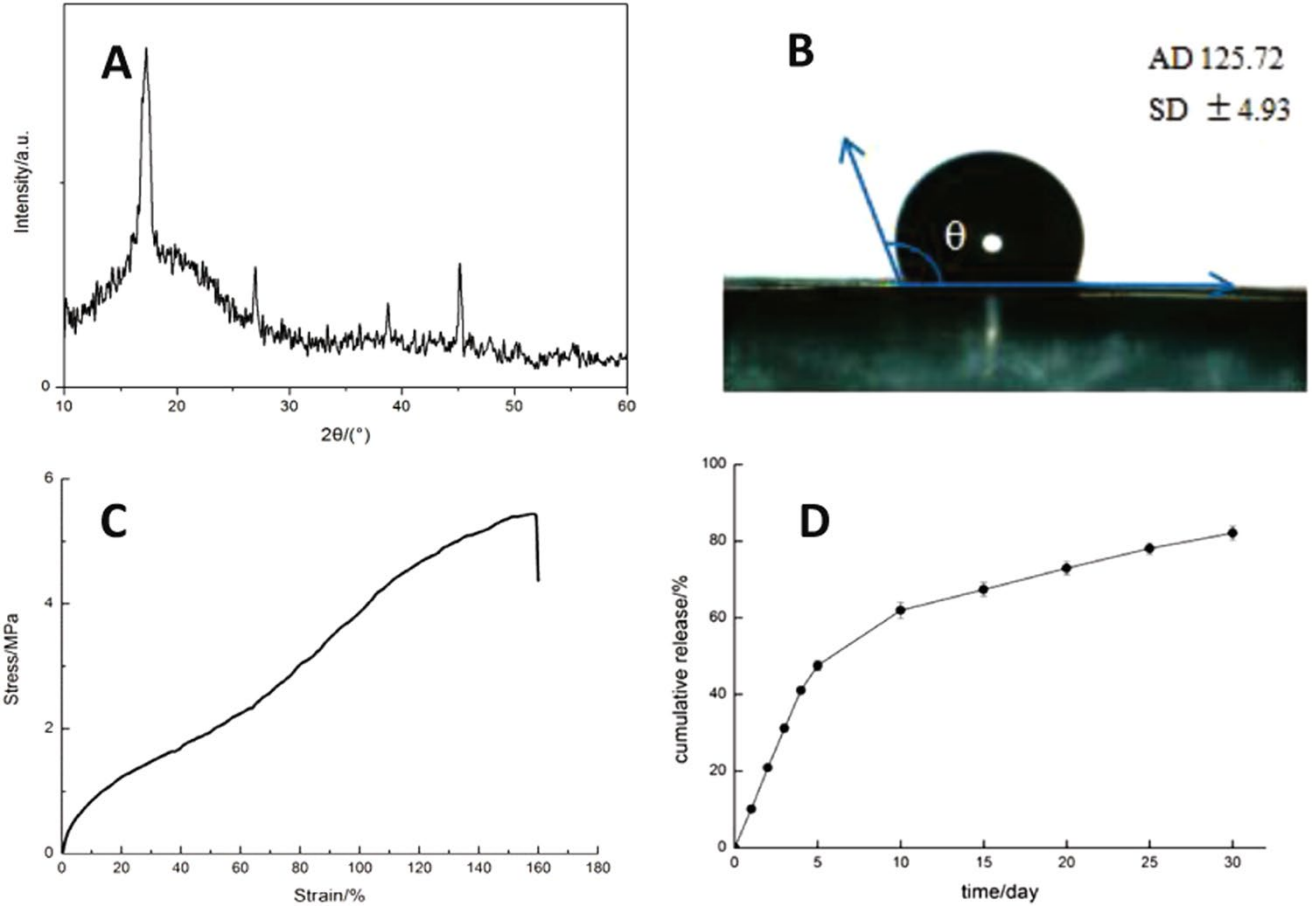
(**A**) XRD analysis of the ICA-SF/PLCL nanofibrous membrane. (**B**) Representative image of water contact angle of membrane at 5 s. (**C**) Stress–strain curve of membrane and (**D**) Release profile of ICA from the drug-loaded membrane.

As shown in the XRD curve of the ICA-SF/PLCL nanofibrous membrane in Fig. 3A, the membrane had new crystalline diffraction peaks appear at about 17.2°, and no other crystalline diffraction peaks were present, demonstrating that the incorporation of ICA had no particular influence on membrane crystallization.

#### Contact angle measurement

The hydrophilicity of the membrane is a pivotal influence on cell adherence and proliferation. The water contact angle was measured on the surface of the ICA-SF/PLCL nanofibrous membrane at 5 s (Fig. 3B) and found to be 125.72 ± 4.93°. Previous studies had confirmed that the water contact angle of the pure PLCL electrospun membrane was 139.57°^28^, so the incorporation of ICA improved the hydrophilicity of the hybrid nanofibrous membrane, which is a benefit to tissue regeneration.

#### Tensile strength tests

The stress–strain curve for the ICA-SF/PLCL nanofibrous membrane in Fig. 3C shows that the tensile strength was 5.54 ± 0.46 MPa, which meets the clinical GBR requirement.

#### Drug release profiles *in vitro*

The ICA release profile of the ICA-SF/PLCL nanofibrous membrane is presented in Fig. 3D. ICA was released in a controlled manner and the entire release period included two stages: an initial burst release stage (about 47.54 ± 0.06% of the ICA released in the first 5 d), followed by a decreasing and constant release stage (ICA release reached 82.09 ± 1.86%), which represents a sustained delivery model.

#### Biodegradation of the nanofibrous membrane *in vitro*

**Figure 4:**
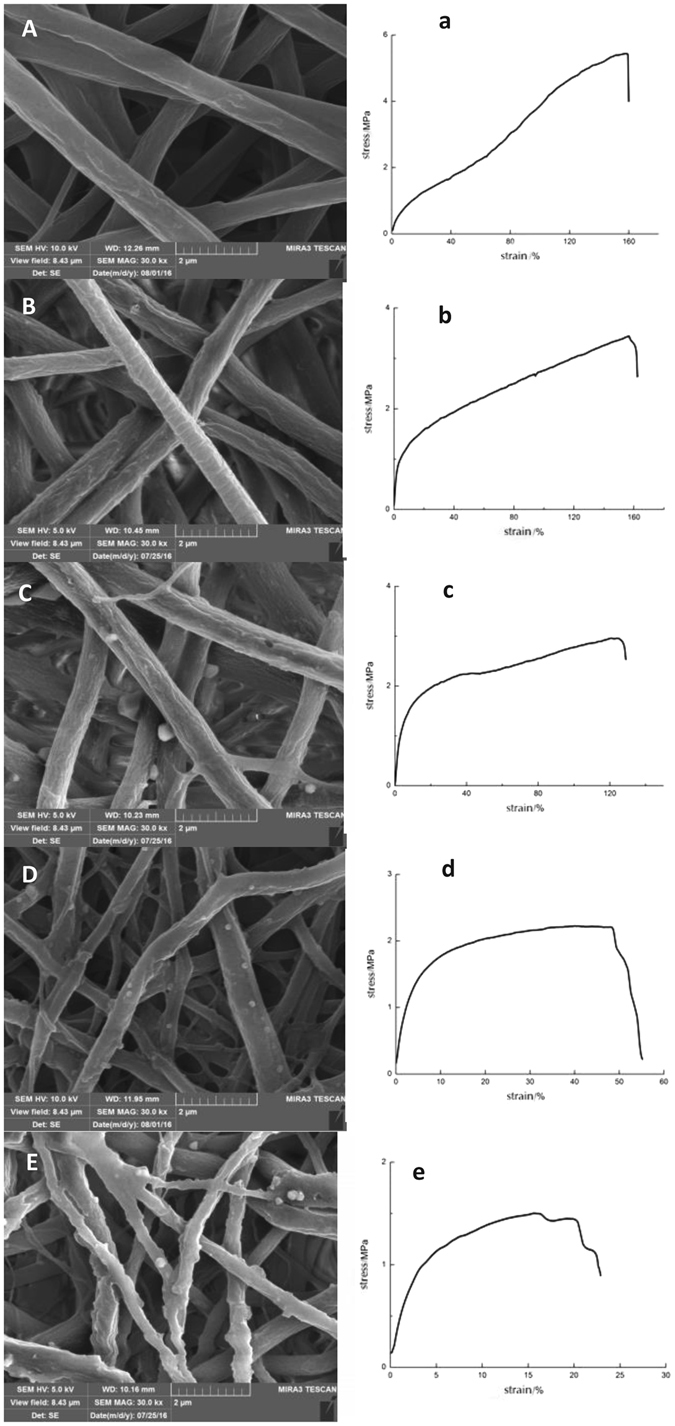
Morphological changes and tensile strength tests of the core–shell structured nanofibrous membrane during PBS immersion. Membranes were incubated for 0 weeks (**A**, a), 1 week (**B**, b), 2 weeks (**C**, c), 4 weeks (**D**, d), and 6 weeks (**E**, e).

The morphological changes and the tensile strength at 0, 1, 2, 4, and 6 weeks after degradation of the membranes *in vitro* were tested (Fig. 4). From the series of SEM images (Fig. 4A–E), we found that for the drug-loaded membrane, along with the drug released, the fibers appeared broken, and the surfaces of the fibers became increasingly rougher. Some collapse could be observed with incubation for 6 weeks (Fig. 4E). The tensile strength tests (Fig. 4a–e) of the membrane showed that with degradation progression, the mechanical properties of the membrane were reduced but adequate to meet the clinical GBR requirement at 6 weeks.

### *In vitro* experiments

#### The activity of released ICA on osteogenic differentiation of BMMSCs

**Figure 5:**
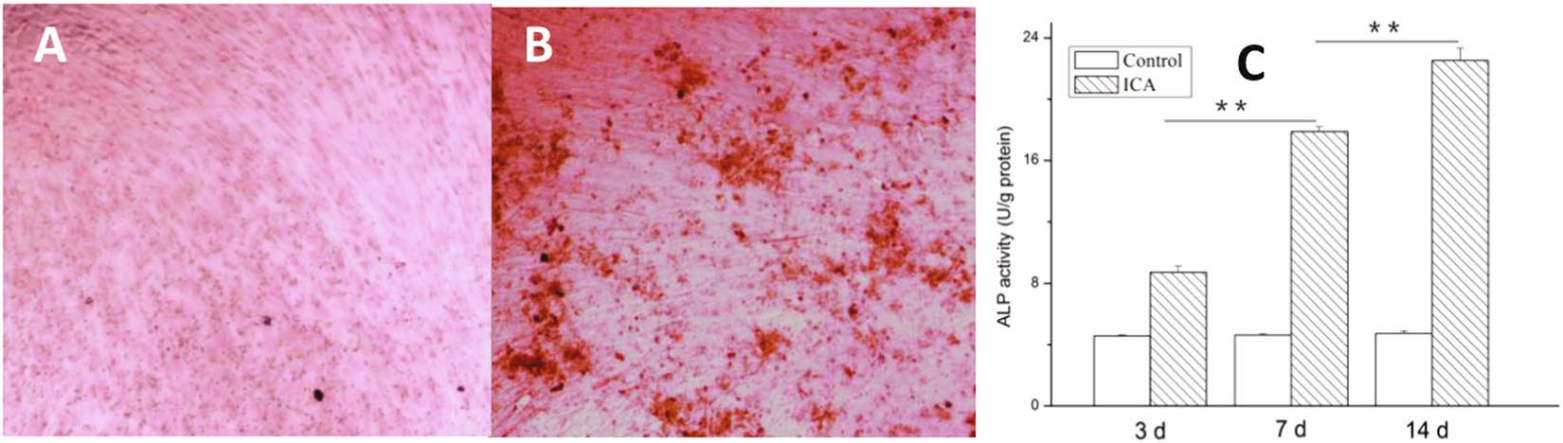
Alizarin Red staining and ALP assay of BMMSCs cultured in ICA-SF/PLCL nanofibrous membrane–released medium. (**A**) Mineralized deposits were not obvious by Alizarin Red staining after 14 d in the control group (x40). (**B**) Mineralized deposits were obvious by Alizarin Red staining after 14 d in the test group (x40). (**C**) ALP activity in the test groups rose at 3, 7, and 14 d. ICA indicates ICA-SF/PLCL drug-loaded nanofibrous membrane group.

Alizarin Red staining and ALP activity assay usually are used as markers of osteogenic differentiation^29^. The BMMSCs were cultured in the medium with ICA-released solution. After 14 d, Alizarin Red staining was evident in obvious mineralized deposits (Fig. 5B), indicating successful osteogenic differentiation of BMMSCs. Figure 5C also shows that the ALP activity in the test groups rose at 3, 7, and 14 d. All of these data suggested that ICA was released from the ICA-SF/PLCL nanofibrous membrane and present at an effective drug concentration in the culture medium for promoting bone regeneration activity.

#### Seeding and morphology of BMMSCs on the membranes

**Figure 6:**
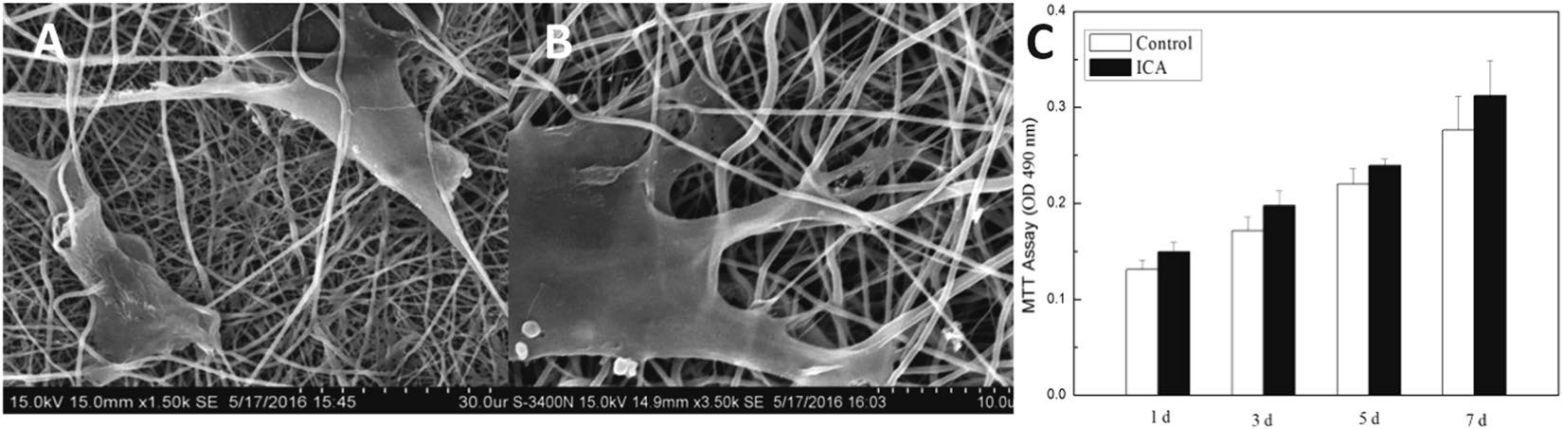
Representative SEM images of BMMSCS–membrane composites after 5 d of culture (Scale bars: (**A)** 30 μm; (**B**) 10 μm) and propagation of BMMSCs cultured on membrane for different periods (**C**). ICA indicates ICA-SF/PLCL drug-loaded nanofibrous membrane group.

The morphological changes of BMMSCs adhered to the membrane after 5 d of culture were observed by SEM (Fig. 6A,B).

#### Cytotoxicity testing of the ICA-SF/PLCL nanofibrous membrane

A drug-loaded membrane is expected to maximize therapeutic activity while minimizing toxic side effects^30^. The optical density results from the MTT assay are shown in Fig. 6C. The values for all groups increased with time at 1, 3, 5, and 7 d, but the relative growth rate showed no significant differences (p > 0.05), indicating that the membrane did not have obvious adverse effects on the viability of BMMSCs. Thus, the membrane seemed to show no cytotoxicity.

### *In vivo* implantation

#### Micro-computed tomography (μ-CT) results

**Figure 7:**
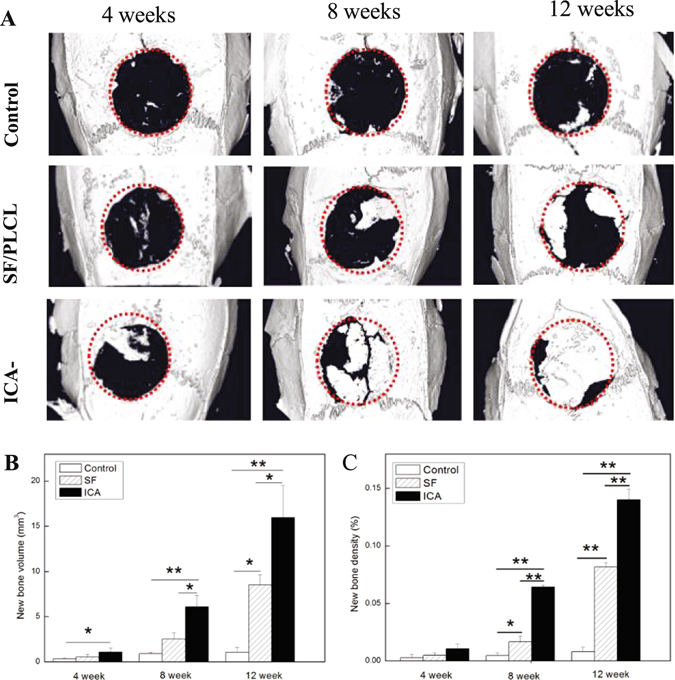
(**A**) μ-CT images of calvarial defects. (**B**) Quantification of percentage of new bone volume and (**C**) density in calvarial defects. *Significantly higher bone formation compared with control (note: *p < 0.05, **p < 0.01. SF indicates SF/PLCL membrane group; ICA indicates ICA–SF/PLCL nanofibrous membrane group; The red dotted line depicts the regions of interest selected for analysis).

To analyze the osteogenic effect of the ICA-SF/PLCL nanofibrous membrane *in vivo*, 8-mm critical-sized rat calvarial complete defects were created. Membranes were implanted and evaluated by μ-CT at 4, 8, and 12 weeks post-implantation. During the experiment, for all animals, no inflammation or necrosis was identified at the implant sites. μ-CT imaging (Fig. 7A) demonstrated more bone formation in the ICA-SF/PLCL nanofibrous membrane group compared with controls at 4, 8, and 12 weeks.

Quantitative analysis of μ-CT images was performed by observing new bone volume and density within the defects (Fig. 7B,C). Results revealed that defects treated with ICA-SF/PLCL nanofibrous membrane showed healing with approximately 1.08 ± 0.48 mm^3^ and 1.04 ± 0.46% new bone volume and density, respectively, after 4 weeks of implantation. The level of new bone formation did not differ significantly between the test groups and the negative controls, and the blank control group showed almost no new bone formation. After 8 weeks of implantation, new bone formation was observed in all groups, compared to the negative control and the blank control groups, the test group showed a significant increase in healing, with 6.09 ± 1.27 mm^3^ bone volume and 6.43 ± 0.17% bone density, respectively.

There were no significant differences between the test group and the blank control group (p > 0.05). At 12 weeks after implantation, although all groups exhibited bone formation, in the test group, it had spread to cover most of the defect region, with volume and density healed of approximately 15.95 ± 3.58 mm^3^ and 14.02 ± 0.93%, showing the fastest and greatest bone formation; bone formation in the blank and negative control groups was limited to the center or at the osteotomy margins. Thus, we confirmed the activity of ICA released from the membrane *in vivo*.

#### Histological analysis

**Figure 8:**
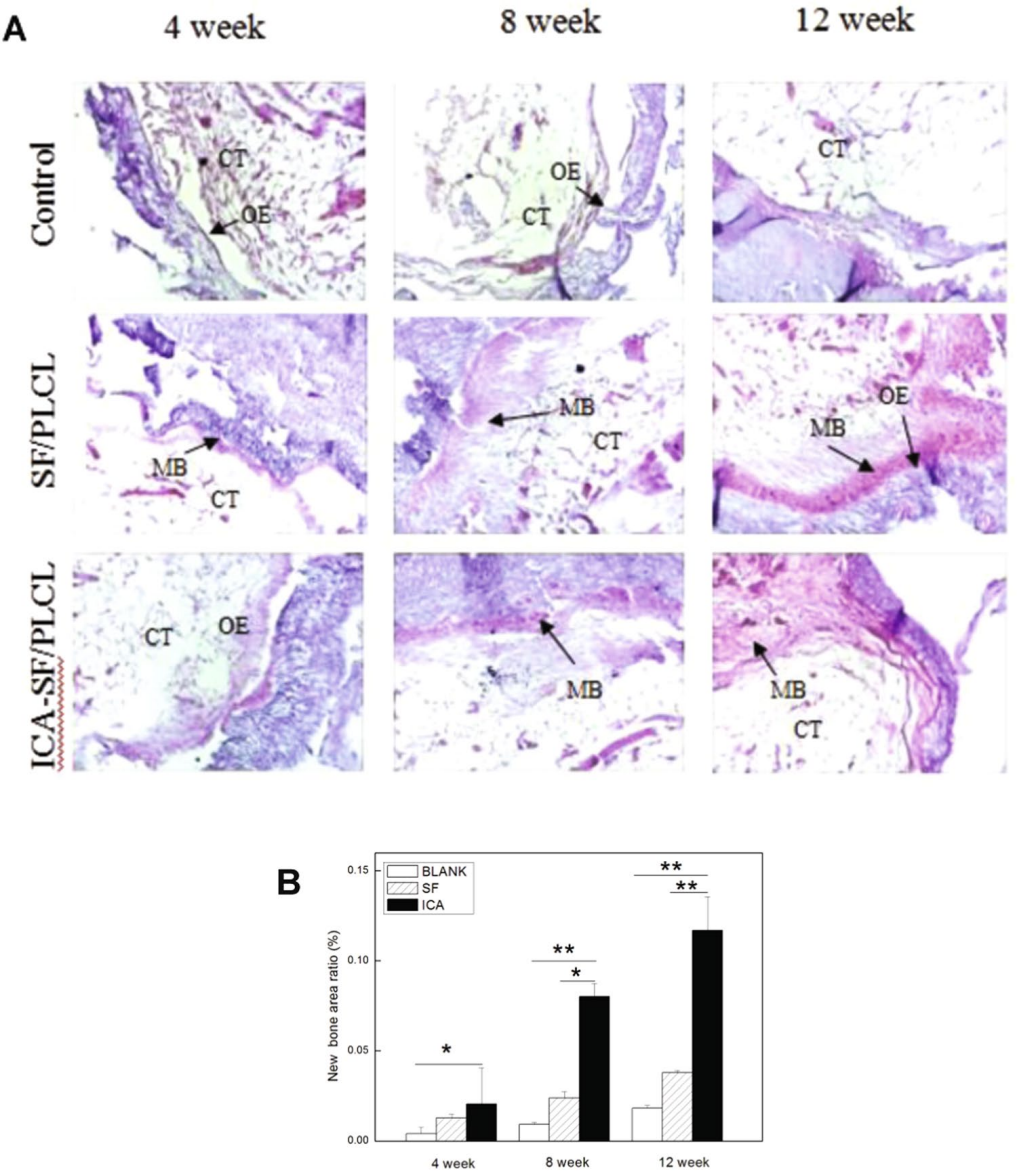
Histological analysis of bone formation in calvarial defects. (**A**) Hematoxylin and eosin staining images of calvarial defects. Scale bar = 0.2 mm. (**B**) Quantification of percentage of new bone area in calvarial defects. *Significantly more bone formation compared with control (*p < 0.05, **p < 0.01). (CT indicates connective tissue; MB indicates mineralized bone matrix; OE indicates osteoectomy edge. SF indicates SF/PLCL membrane group; ICA indicates ICA-SF/PLCL nanofibrous membrane group).

The new bone formation was further characterized by histological examination at 4, 8, and 12 weeks post-surgery, and the ratio of new bone area to the total defect area was calculated (Fig. 8B). At 4 weeks post-operation, some new bone was observed in both the test and negative control groups, but in the blank control group, the defects were filled with soft tissue and minimal bone formation. At 8 weeks post-operation, the test group exhibited faster and more effective osteogensis than the negative or blank control group. In the blank control group, a mass of fibrous connective tissue was observed. At 12 weeks post-operation, the new bone area ratio was significantly higher in the test group than in other groups. In the negative control group, new bone filled the defect but was mixed with fibro-adipose tissue. The blank control group had only some limited fibrous connective tissue.

## Discussion

Our previous work and that of others showed that during the coaxial electrospinning process, the flow rate, receiving distance, electric current, and other spinning parameters can influence the diameter of the fiber. All the parameters chosen were optimized by our previous work. The results showed that the membrane fabricated was nano-scaled and TEM showed the fiber exhibited an obvious core–shell structure. Evaluation of the position of the diffraction peaks by XRD analysis can allow detection of the crystal structure of the matter^31^. The conformation study had yielded crystalline diffraction peaks of silk fibroin (Silk I) located at 12.2°, 19.7°, 24.7° and 28.2°^32^, and the characteristic peaks of pure PLCL electrospun membrane located at about 23.7°^33^, respectively. Silk I has a poor conformational regularity and can be destroyed easily while Silk II is characterized by a β fold, has a relatively good conformational regularity, and is relatively stable. Crosslinked by 2.5% GTA solution, the structure of the ICA-SF/PLCL nanofibrous membrane had changed the α helix into a β fold, and the crystallinity was reinforced^34^. The structure of SF is similar to that of the extracellular matrix, it is a good candidate as a cell scaffold among natural polymers^17^, but it is breakable. In this study, added PLCL greatly improved the mechanical properties of the nanofibrous membrane. Data confirmed that this ICA-SF/PLCL membrane had good morphological characterization and suitable physical, chemical and mechanical properties.

It is essential to supply an effective drug concentration, and to circumvent systemic side effects, a drug delivery system to release drugs into target sites at a steady rate is desired^14^. The formation of a distinct boundary can be theoretically analyzed as described previously^25, 28^. During the coaxial electrospinning process, under the influence of high voltage and electric force, the time of shell/core layer spinning solution bending instability is much shorter than that of diffusion spreading. Simultaneously, ICA, the bigger molecule, is inclined to move toward the inner part of the nanofiber and be encapsulated in the fiber. Therefore, coaxial electrospinning can realize drug loading and achieve the purpose of sustained and controlled local drug delivery. The mechanism of ICA released in a controlled manner and the two stages entire release period is explained mainly by two considerations^35, 36^, diffusion and degradation of the polymer. The drug is initially released from the membrane by diffusion in the fiber surface. Subsequently, with the scouring effect of dissolution medium on the fiber surface, once the membrane was incubated in the release media, some small pores appeared on the surface of the fibers. The drug was released through the pores, resulting in the subsequent slow and relatively steady release rate. Naturally, drug diffusion into the dissolution medium is also affected by the shell thickness. Under the effects of these factors, the ICA-SF/PLCL nanofibrous membrane showed a steady and sustained drug release.

Implantable material is desired to degrade as the replacement produced by natural tissue develops simultaneously^37^. Material degradation speed is an important factor to consider in tissue engineering^38–40^. If the degradation of the scaffold is too rapid, the cells would not proliferate well and secrete sufficiently new matrix^38^. However, the degradation should not be too slower than new bone formation otherwise the rest materials will influence the homogeneity and biological function of the regenerated bone^41^. GBR require a membrane not to degraded too fast to maintain a stable space beneath for new bone formation. In the present study, the fibers became increasingly rougher and the tensile strength decreased within 6 weeks along with the drug released. The membrane’s morphology changed but exactly meeting the clinical GBR requirement at 6 weeks.

The nano-scale structure of the fibers is similar to the natural extracellular matrix. It has a high surface-to-volume ratio and could improve cell attachment^24, 42^. Simultaneously, fibers cross-formed pore structures that allowed sufficient permeability to nutrients and gas exchange for the regenerated tissue^43^. The morphology of combination of BMMSCs and fibers exhibited a biocompatible property of the membrane. SEM established that cells are tightly anchored along the fibers of the membrane, with cytoplasmic processes emitted and spread in all directions, suggesting that the membrane offered an ideal scaffold for cell adhesion and growth with good biocompatibility; cell proliferation *in vitro* was similar to that *in vivo*. Moreover, we found that the cells proliferated on the surface without invading the internal part of the membrane, showing excellent barrier function which is the main point of the current GBR technology.

The present study demonstrated that ICA-SF/PLCL fibers can significantly promote the osteogenesis of BMMSCs *in vitro* and repair bone defect *in vivo*. As the only active factor, ICA could be successfully delivered and released in a controlled manner. Its contribution to osteogenesis have been studied by many researchers (some were mentioned in the part of introduction). Our team has been concerned about the role of ICA in promoting osteogenesis and our previous studies have confirmed that ICA can significantly promote the proliferation and osteogenic differentiation abilities of human periodontal ligament stem cells both *in vitro* and *in vivo* ^12^. Zhang studied the effect of icariin on the activity of osteoblasts and osteoclasts in the co-culture system. The results showed that ICA could promote the osteogenic activity of mouse osteoblast of MC3T3-E1 cells and inhibit the mouse osteoclast activity of RAW264.7 cells, NF-κB gene and protein expression of osteoclast RAW264.7 were significantly decreased^44^.

Liming Xue found that twenty three proteins in bone femur, and 8 metabolites in serum, were significantly altered and identified in ICA treatment group, Related to bone remodeling, Ca^2+^ signaling *et al*. The cytoskeleton of both osteoblast and osteoclast in icariin-treated group were enhanced while the inhibition of collagenolytic activity of cathepsin K, mRNA expression of MMP-9 and protein expression of ERK in osteoclast were not detected. Their conclusion was that icariin is multi-targeting compounds for treating bone remodeling. Its function on inhibition of osteoclasts differentiation mainly through OPG/RANKL signal and MAPK signal. ICA also could reduce the number and activity of osteoclasts^45^.

Other researchers demonstrated that ICA could activate the proliferation and differentiation of osteoblast and inhibit the differentiation and bone resorption enzymes to regulate bone resorption^46–49^. All these results presented are consistent with the findings in our study. ICA could be a strong positive inducer for bone regeneration by regulating both osteoblast and osteoclast.

## Conclusion

We successfully fabricated an ICA-SF/PLCL nanofibrous membrane by coaxial electrospinning as an osteoinductive GBR membrane to promote bone regeneration. ICA was incorporated into the nanofiber without loss of structural integrity or change in functionality. *In vitro* osteogenic differentiation and *in vivo* implantation experiments indicated that the membrane could effectively deliver ICA in a sustained manner and significantly promote bone regeneration with no cytotoxicity. Thus, coaxial electrospun technology could be an effective way to fabricate membranes for osteoinductive GBR and that the ICA-SF/PLCL nanofibrous membrane is a promising biomaterial for GBR in dental implants.

## Electronic supplementary material

Supplementary Information

## References

[1] Albrektsson T (1988). Osseointegrated oral implants. A Swedish multicenter study of 8139 consecutively inserted Nobelpharma implants. J Periodontol..

[2] Verhoeven JW, Cune MS, de Putter C (2000). Reliability of some clinical parameters of evaluation in implant dentistry. J Oral Rehabil..

[3] Hämmerle CH, Karring T (1998). Guided bone regeneration at oral implant sites. Periodontol 2000..

[4] Danesh-Sani SA, Tarnow D, Yip JK, Mojaver R (2017). The influence of cortical bone perforation on guided bone regeneration in humans. Int J Oral Maxillofac Surg..

[5] Lu S (2015). A novel silk fibroin nanofibrous membrane for guided bone regeneration: a study in rat calvarial defects. Am J Transl Res..

[6] Soldatos NK (2017). Limitations and options using resorbable versus nonresorbable membranes for successful guided bone regeneration. Quintessence Int..

[7] Cho WJ (2009). Hydrophilized polycaprolactone nanofiber mesh-embedded poly(glycolic-co-lactic acid) membrane for effective guided bone regeneration. J Biomed Mater Res A..

[8] Li, X. A., Ho, Y. S., Chen, L. & Hsiao, W. L. The Protective Effects of Icariin against the Homocysteine-Induced Neurotoxicity in the Primary Embryonic Cultures of Rat Cortical Neurons. *Molecules*. **21**, pii: E1557 (2016). 10.3390/molecules21111557 PMC6274412 27879670

[9] Liu B (2011). Neuroprotective effects of icariin on corticosterone-induced apoptosis in primary cultured rat hippocampal neurons. Brain Res..

[10] Zhang G, Qin L, Shi Y (2007). Epimedium-derived phytoestrogen flavonoids exert beneficial effect on preventing bone loss in late postmenopausal women: a 24-month randomized, double-blind and placebo-controlled trial. Bone Miner Res..

[11] Zhang G, Qin L, Shi Y (2007). Epimedium-derived phytoestrogen flavonoids exert beneficial effect on preventing bone loss in late postmenopausal women: a 24-month randomized, double-blind and placebo-controlled trial. J Bone Miner Res..

[12] Qin Z (2015). Effects of Icariin promotion on proliferation and osteogenic differentiation of human periodontal ligament stem cells. Hua Xi Kou Qiang Yi Xue Za Zhi..

[13] Vysloužilová L (2016). Needleless coaxial electrospinning: A novel approach to mass production of coaxial nanofibers. Int J Pharm..

[14] Li H (2010). Controlled release of PDGF-bb by coaxial electrospun dextran/poly(L-lactide-co-epsilon-caprolactone) fibers with an ultrafine core/shell structure. J Biomater Sci Polym Ed..

[15] Kim JY, Yang BE, Ahn JH, Park SO, Shim HW (2014). Comparable efficacy of silk fibroin with the collagen membranes for guided bone regeneration in rat calvarial defects. J Adv Prosthodont..

[16] Yucel T, Lovett ML, Kaplan DL (2014). Silk-based biomaterials for sustained drug delivery. J Control Release..

[17] Kundu B, Rajkhowa R, Kundu SC, Wang X (2013). Silk fibroin biomaterials for tissue regenerations. Adv Drug Deliv Rev..

[18] Tu Y (2017). Fabrication of nano-hydroxyapatite/chitosan membrane with asymmetric structure and its applications in guided bone regeneration. Biomed Mater Eng..

[19] Saulacic N, Fujioka-Kobayashi M, Kobayashi E, Schaller B, Miron RJ (2017). Guided bone regeneration with recombinant human bone morphogenetic protein 9 loaded on either deproteinized bovine bone mineral or a collagen barrier membrane. Clin Implant Dent Relat Res..

[20] Ma S (2016). Asymmetric Collagen/chitosan Membrane Containing Minocycline-loaded Chitosan Nanoparticles for Guided Bone Regeneration. Sci Rep..

[21] Won JY (2016). Evaluation of 3D printed PCL/PLGA/*β*-TCP versus collagen membranes for guided bone regeneration in a beagle implant model. Biomed Mater..

[22] Zhu H (2013). Biological activity of a nanofibrous barrier membrane containing bone morphogenetic protein formed by core-shell electrospinning as a sustained delivery vehicle. J Biomed Mater Res B Appl Biomater..

[23] Yin LH (2014). A research on the performance of SF/COL/PLCL electrospun three-dimensional nanofiber scaffold. J Funct Mater..

[24] Yin L (2017). Physicochemical and biological characteristics of BMP-2/IGF-1-loaded three-dimensional coaxial electrospun fibrous membranes for bone defect repair. J Mater Sci Mater Med..

[25] Amschler K, Erpenbeck L, Kruss S, Schön MP (2014). Nanoscale integrin ligand patterns determine melanoma cell behavior. ACS Nano..

[26] Su Y (2012). Controlled release of bone morphogenetic protein 2 and dexamethasone loaded in core-shell PLLACL-collagen fibers for use in bone tissue engineering. Acta Biomater..

[27] Nadima A, Khorasania SN, Kharazihab M, Davoodi SM (2017). Design and characterization of dexamethasone-loaded poly (glycerol sebacate)-poly caprolactone/gelatin scaffold by coaxial electro spinning for soft tissue engineering. Mater Sci Eng C Mater Biol Appl..

[28] Qian C, Zhu C, Yu W, Jiang X, Zhang F (2015). High-Fat Diet/Low-Dose Streptozotocin-Induced Type 2 Diabetes in Rats Impacts Osteogenesis and Wnt Signaling in Bone Marrow Stromal Cells. PLoS One..

[29] Zhu L, Liu X, Du L, Jin Y (2016). Preparation of asiaticoside-loaded coaxially electrospinning nanofibers and their effect on deep partial-thickness burn injury. Biomed Pharmacother..

[30] Singhal P, Small W, Cosgriff-Hernandez E, Maitland DJ, Wilson TS (2014). Low density biodegradable shape memory polyurethane foams for embolic biomedical applications. Acta Biomater..

[31] Kim J, Tanner K (2016). Three-Dimensional Patterning of the ECM Microenvironment Using Magnetic Nanoparticle Self Assembly. Curr Protoc Cell Biol..

[32] Yang BY, Cao Y, Qi FF, Li XQ, Xu Q (2015). Atrazine adsorption removal with nylon6/polypyrrole core-shell nanofibers mat: possible mechanism and characteristics. Nanoscale Res Lett..

[33] Ji X (2013). Coaxially electrospun core/shell structured poly(L-lactide) acid/chitosan nanofibers for potential drug carrier in tissue engineering. J Biomed Nanotechnol..

[34] Fetterly, K, Greason, K, Mathew, V. Balloon Valvuloplasty to Predict X-ray Projection Angles that are Perpendicular to Cardiovascular Structures: A TAVI Patient Feasibility Study. *Catheter Cardiovasc Interv*. **29**, doi:10.1002/ccd.26879 (2016). 10.1002/ccd.26879 27896912

[35] Liu Q (2014). Materials research of SF/COL/PLCL and SF/COL/PLLA electrospun three-dimensional nanofiber scaffold. Journal of Functional Materials..

[36] Bigi A, Cojazzi G, Panzavolta S, Rubini K, Roveri N (2001). Mechanical and thermal properties of gelatin films at different degrees of glutaraldehyde crosslinking. Biomaterials..

[37] Fu W (2014). Electrospun gelatin/PCL and collagen/PLCL scaffolds for vascular tissue engineering. Int J Nanomedicine..

[38] Gilbert TW, Stewart-Akers AM, Badylak SF (2007). A quantitative method for evaluating the degradation of biologic scaffold materials. Biomaterials..

[39] Sung HJ, Meredith C, Johnson C, Galis ZS (2004). The effect of scaffold degradation rate on three-dimensional cell growth and angiogenesis. Biomaterials..

[40] Alsberg E (2003). Regulating bone formation via controlled scaffold degradation. J Dent Res..

[41] Gong YY (2011). A sandwich model for engineering cartilage with acellular cartilage sheets and chondrocytes. Biomaterials..

[42] Mi R, Liu Y, Chen X, Shao Z (2016). Structure and properties of various hybrids fabricated by silk nanofibrils and nanohydroxyapatite. Nanoscale..

[43] Teo BK (2013). Nanotopography modulates mechanotransduction of stem cells and induces differentiation through focal adhesion kinase. ACS Nano..

[44] Zhang S (2017). Icariin influences adipogenic differentiation of stem cells affected by osteoblast-osteoclast co-culture and clinical research adipogenic. Biomed Pharmacother..

[45] Xue L (2016). Comparative proteomic and metabolomic analysis reveal the antiosteoporotic molecular mechanism of icariin from Epimedium brevicornu maxim. J Ethnopharmacol..

[46] Ma HP (2011). Icariin is more potent than genistein in promoting osteoblast differentiation and mineralization *in vitro*. J Cell Biochem..

[47] Ma XN (2013). Icariin induces osteoblast differentiation and mineralization without dexamethasone *in vitro*. Planta Med..

[48] Sun P (2013). An inhibitor of cathepsin K, icariin suppresses cartilage and bone degradation in mice of collagen-induced arthritis. Phytomedicine..

[49] Zhang J, Song J, Shao J (2015). Icariin attenuates glucocorticoid-induced bone deteriorations, hypocalcemia and hypercalciuria in mice. Int J Clin Exp Med..

