# Supplementary material for: The fabrication of an ICA-SF/PLCL nanofibrous membrane by coaxial electrospinning and its effect on bone regeneration in vitro and in vivo
Source: Sci Rep. 2017 Aug 17;7:8616. doi: 10.1038/s41598-017-07759-8 (PMC5561113; doi:10.1038/s41598-017-07759-8)
Supplement: Supplementary file 1 — Supplementary Information [file 41598_2017_7759_MOESM1_ESM.pdf]

The fabrication of an ICA-SF/PLCL nanofibrous membrane by coaxial electrospinning and its effect on bone regeneration in vitro and in vivo

Lihua Yin[1*], Kaijuan Wang[2], Xiaoqin Lv[2], Rui Sun[2], Shaohua Yang[2], Yujie Yang[2], Yanyun Liu[2], Jiatao Liu[2], Jing Zhou[2], Zhanhai Yu[1]

[1] Department of Oral Implantology, School/Hospital of Stomatology, Lanzhou University, Lanzhou, China, 730000.
[2] School/Hospital of Stomatology, Lanzhou University, Lanzhou, China, 730000.
Email: *yinlh@lzu.edu.cn

Reprint requests and correspondence to Lihua Yin, DDS, Ph.D. Associate professor, Director, yinlh@lzu.edu.cn

Supplementary material

BMMSCs Identification

The immunophenotype of monolayer and single colony-derived adherent cells from SD rat bone marrow was determined by flow cytometry using the antibodies listed in Table S1. As shown in Table S1, BMMSCSs should stained positively for CD44 (Biolegend, USA) and CD90 (BD, USA) and negatively for CD34 (Multi Sciences, Hangzhou, China) and CD45 (BD, USA).

Table S1 The result of flow cytometry：(a) CD34, (b) CD45, (c) CD44 and (d) CD90.

Antigen	Reactivity
CD34	-
CD44	+
CD45	-
CD90	+

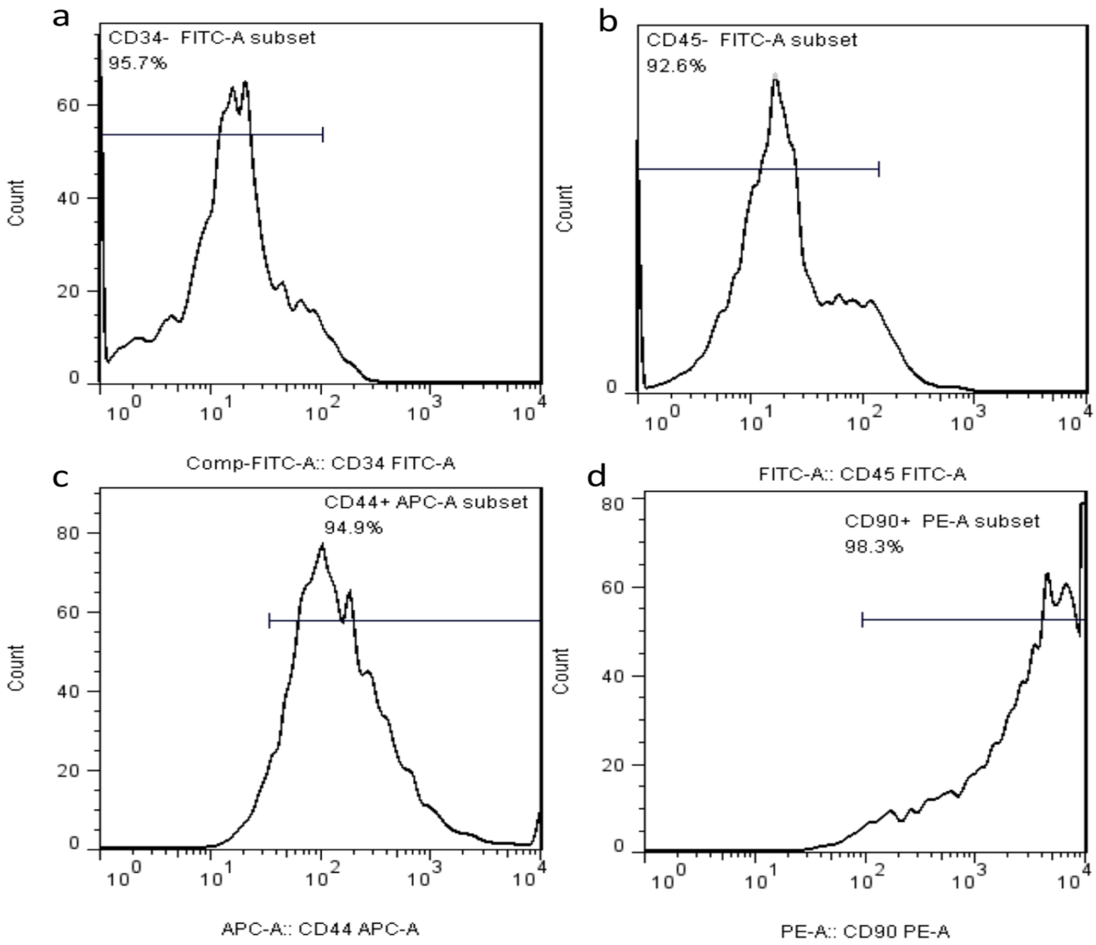

Fig.S1 The results of flow cytometry：(a) CD34, (b) CD45, (c) CD44 and (d) CD90.

As shown in Fig. S1, the negative rates of CD34 and CD45 were 95.7% and 92.6% respectively, and the positive rates of CD44 and CD90 were 94.9% and 98.3% respectively, which suggested that BMMCs expressed CD44 and CD90 but not CD34 and CD45. Therefore, the cells used in the experiment can be identified as bone marrow mesenchymal stem cells.
